# Multimodal monitoring of cerebral perfusion in carotid endarterectomy patients: a computational fluid dynamics study

**DOI:** 10.3389/fneur.2024.1455401

**Published:** 2024-12-05

**Authors:** Lei Guo, Jun Zhang, Kai Lv, Xiong Li, Meiling Guo, Chunling Li

**Affiliations:** ^1^Department of Neurosurgery, Sichuan Academy of Medical Sciences and Sichuan Provincial People’s Hospital, University of Electronic Science and Technology of China, Chengdu, China; ^2^Department of Neurology, Xindu District People's Hospital of Chengdu, Chengdu, China; ^3^School of Mechanical and Electrical Engineering, University of Electronic Science and Technology of China, Chengdu, China

**Keywords:** carotid artery stenosis, carotid endarterectomy, multimodal monitoring, cerebral hyperperfusion, computational fluid dynamics

## Abstract

**Objective:**

To evaluate postoperative cerebral perfusion changes and their influencing factors in carotid endarterectomy (CEA) patients by integrating multimodal monitoring methods, including cerebral regional oxygen saturation (rSO_2_), carotid ultrasound (CU), computed tomographic angiography (CTA), and computed tomographic perfusion imaging (CTP), with computational fluid dynamics (CFD) assessment.

**Methods:**

We conducted a cohort study on patients with internal carotid artery (ICA) stenosis undergoing CEA at our institution. Pre- and postoperative assessments included CU, CTA, CTP, and rSO_2_ monitoring. Hemodynamic parameters recorded were mean flow velocity (MFV), peak systolic velocity (PSV), end diastolic velocity (EDV), resistance index (RI), rSO_2_, and cerebral blood flow (CBF). CFD quantified the total pressure (TP), wall shear stress (WSS), wall shear stress ratio (WSSR), and translesional pressure ratio (PR) of the ICA. Pearson correlation was used to analyze factors influencing cerebral perfusion changes. Multivariate logistic regression identified risk factors for cerebral hyperperfusion (CH). The predictive value of multimodal and single-modality monitoring for CH was evaluated using ROC curve analysis.

**Results:**

Fifty-six patients were included, with nine developing postoperative CH. CU showed significant reductions in MFV, PSV, EDV, and RI of the ICA (*p* < 0.001). Ipsilateral rSO_2_ increased significantly (*p* = 0.013), while contralateral rSO_2_ showed no significant change (*p* = 0.861). CFD revealed significant decreases in TP, WSS, and WSSR (*p* < 0.001), along with a significant increase in PR (*p* < 0.001). Pearson analysis indicated that change rate of CBF (*Δ*CBF) positively correlated with ΔPR and ΔrSO_2,_ and negatively correlated with ΔTP, ΔWSS, and Δ WSSR. Multivariate logistic regression identified preoperative WSSR (pre-WSSR) and ΔPR as risk factors for CH following CEA. Combined ΔPR, ΔrSO_2,_ ΔMFV, and pre-WSSR had higher sensitivity and specificity than single-modality monitoring for predicting CH.

**Conclusion:**

CFD-based multimodal monitoring effectively identified cerebral perfusion changes and risk factors for CH in CEA patients, with superior predictive accuracy compared to single-modality methods. Nevertheless, further validation is necessary to establish its clinical utility.

## Introduction

Carotid artery stenosis is a notable contributor to cerebrovascular disease, with a cumulative stroke risk of approximately 15% at 1 year, 26% at 2 years, and 30% at 5 years for severe stenosis (1). Over the past decades, clinical trials have investigated the effectiveness and significant advantages of carotid endarterectomy (CEA) in preventing long-term strokes among patients with carotid artery stenosis (2). However, perioperative complications such as stroke, hemodynamic disturbances, and cranial nerve injuries may arise following CEA, particularly cerebral hyperperfusion (CH) impairment, which is a fatal complication during the postoperative period and significantly impacts long-term patient survival and neurological recovery (3, 4). CH pathophysiology involves impaired cerebral autoregulation, postoperative hypertension, ischemia–reperfusion injury, and structural vascular damage. Blood pressure fluctuations around surgery disrupt autoregulation, causing abnormal cerebral blood flow increases. Chronically ischemic, weakened vessels face a higher hemorrhage risk. Ischemia–reperfusion also produces reactive oxygen species, worsening vascular damage (5). Early identification of risk factors associated with CH post-CEA is essential for guiding future strategies to reduce complications and enhance the safety of CEA procedures.

Currently, clinical methods used to monitor perioperative cerebral hemodynamics include computed tomographic angiography (CTA), computed tomographic perfusion imaging (CTP), carotid ultrasound (CU), transcranial Doppler ultrasound (TCD), and regional cerebral oxygen saturation (rSO_2_) monitoring. Each method possesses distinct advantages and limitations for assessing perioperative cerebral hemodynamics (6). CTP provides a rapid and minimally invasive evaluation of cerebral perfusion and is highly sensitive for diagnosing CH, but it may not be suitable for early warning of CH and requires caution in patients with contrast allergies or renal impairment (7). CU is non-invasive and quickly assesses blood flow parameters, but it is restricted to evaluating the carotid arteries. TCD enables real-time monitoring of intracranial blood flow but lacks the ability to directly measure perfusion volume (8). rSO_2_ monitoring provides real-time feedback on cerebral oxygen supply–demand balance, yet its use for cerebral perfusion assessment remains controversial due to monitoring range limitations and external influences (9). Some researchers consider a decrease in rSO_2_ exceeding 20% from baseline in awake patients indicative of inadequate tissue perfusion (10), while others argue that rSO2 values below 50% are clinically significant (11, 12).

Recent advancements in image acquisition technology and refined post-processing methods within the field of biomedical engineering have elevated computational fluid dynamics (CFD) to a prominent tool for the quantitative analysis of hemodynamics (13). CFD enables the visualization and assessment of blood flow in diverse vascular pathologies, providing primary parameters such as flow rate and velocity as well as more advanced metrics including wall shear stress (WSS), wall shear stress ratio (WSSR), total pressure (TP), pressure ratio (PR) across lesions, and resistance index (RI) (14). Studies have demonstrated a correlation between changes in WSS and the progression of atherosclerosis in both animal and human subjects (15). Nevertheless, the heterogeneity inherent in using solely CFD models impedes their widespread clinical application. Therefore, this study integrated multimodal monitoring techniques with CFD analysis to systematically assess dynamic changes in cerebral perfusion during the perioperative period of CEA patients and their potential influencing factors.

## Methods

### Study participants

This cohort study, conducted at a single center, enrolled patients diagnosed with carotid artery stenosis who underwent CEA at Sichuan Provincial People’s Hospital from January 2023 to July 2024. Inclusion criteria included: (1) age ≥ 18 years; (2) diagnosis of carotid artery stenosis according to the North American Symptomatic Carotid Endarterectomy Trial (NASCET) criteria, meeting CEA treatment guidelines: asymptomatic stenosis exceeding 70% or symptomatic stenosis with a transient ischemic attack or ipsilateral ischemic stroke within the previous 6 months and stenosis ranging from 50 to 99% (16); and (3) informed consent signed by patients and their families. Exclusion criteria were: (1) intracranial hemorrhage within the past year; (2) major stroke or myocardial infarction within the last 30 days; (3) progressive stroke within the previous 3 months; (4) untreated or untreatable large aneurysms; (5) chronic total occlusion without significant cerebral ischemia; (6) coagulopathy; (7) anesthesia intolerance; (8) severe cardiac, pulmonary, hepatic, or renal dysfunction; and (9) severe dementia. The study protocol is registered at ClinicalTrials.gov (ID: NCT06294496) and received approval from the ethics committee of Sichuan Provincial People’s Hospital (Approval No. [2023] 273).

### Clinical data collection

Data on patient characteristics and clinical outcomes were collected prospectively. Baseline data included sex, age, history of smoking, alcohol use, hypertension, diabetes, hyperlipidemia, and coronary artery disease. Serum lipid levels encompassed total cholesterol (TC), triglycerides (TG), high-density lipoprotein cholesterol (HDL-C), and low-density lipoprotein cholesterol (LDL-C). Characteristics of carotid stenosis included location (left ICA or right ICA), symptomatic or asymptomatic ICA, and severity of stenosis. Medical resources assessed were length of hospital stay and hospitalization expenses.

All patients underwent CU, carotid CTA, cerebral CTP imaging, and rSO_2_ monitoring within 3 days before and after surgery. Perioperative CU parameters, including mean flow velocity (MFV), peak systolic velocity (PSV), end diastolic velocity (EDV), and resistance index (RI), were recorded. The MFV change rate was calculated as ΔMFV = [(post-MFV − pre-MFV) / pre-MFV] × 100%. CT perfusion parameters included ipsilateral and contralateral cerebral blood flow (CBF) and relative cerebral blood flow (rCBF) (17). Regional cerebral oxygen saturation (rSO_2_) was measured using a cerebral and regional tissue oxygen saturation monitor (01-06-X100, Jiangxi Iludeli Medical Technology Co., Ltd.), positioned 1 cm lateral to the forehead midline and 1–2 cm above the supraorbital margin. rSO_2_ readings were taken every minute, three times in a row, and averaged. The rSO_2_ variation rate was calculated as ΔrSO_2_ = [(post-rSO_2_ − pre-rSO_2_) / pre-rSO_2_] × 100%.

### CEA procedure

All CEA procedures were conducted by experienced neurosurgeons utilizing an eversion technique while the patient was under general anesthesia. The carotid sheath was exposed by making a longitudinal incision along the anterior border of the sternocleidomastoid muscle. The common carotid artery (CCA), ICA, external carotid artery (ECA), and their respective branches were visualized and temporarily occluded. A 3 mm incision was created at the origin of the ICA, followed by its division and eversion. Atherosclerotic plaques and thickened intima were then meticulously dissected from the ICA. The residual debris was cleared, followed by continuous suturing of the vessels using non-traumatic vascular sutures, and subsequently, the ECA, CCA, and ICA were sequentially reopened.

### Perioperative management and outcome definition

Institutional standards were followed during perioperative management (18). Patients undergoing CEA received 100 mg of aspirin daily for at least a week before surgery. After surgery, patients were treated in the neuro-intensive care unit for at least 24 h, monitoring consciousness, limb sensation, and muscle strength. Postoperative systolic pressure was maintained within the range of 120–140 mmHg, and random blood glucose levels were controlled to stay below 11 mmol/L. Aspirin therapy continued for 1–3 months after surgery, with duration adjusted according to each patient’s condition.

The primary objective of this study was to assess the utility and validity of multimodal cerebral perfusion monitoring techniques, with alterations in cerebral perfusion quantified by changes in cerebral blood flow (ΔCBF). The ΔCBF was determined using the formula: ΔCBF = [(post-CBF − pre-CBF) / pre-CBF] × 100%. Additionally, a secondary aim was to identify factors influencing cerebral hyperperfusion (CH) following CEA, defined as a postoperative increase of 100% or more in ΔCBF within the ipsilateral middle cerebral artery territory compared to preoperative levels.

### CFD modeling and quantification of hemodynamic characteristics

**Figure 1:**
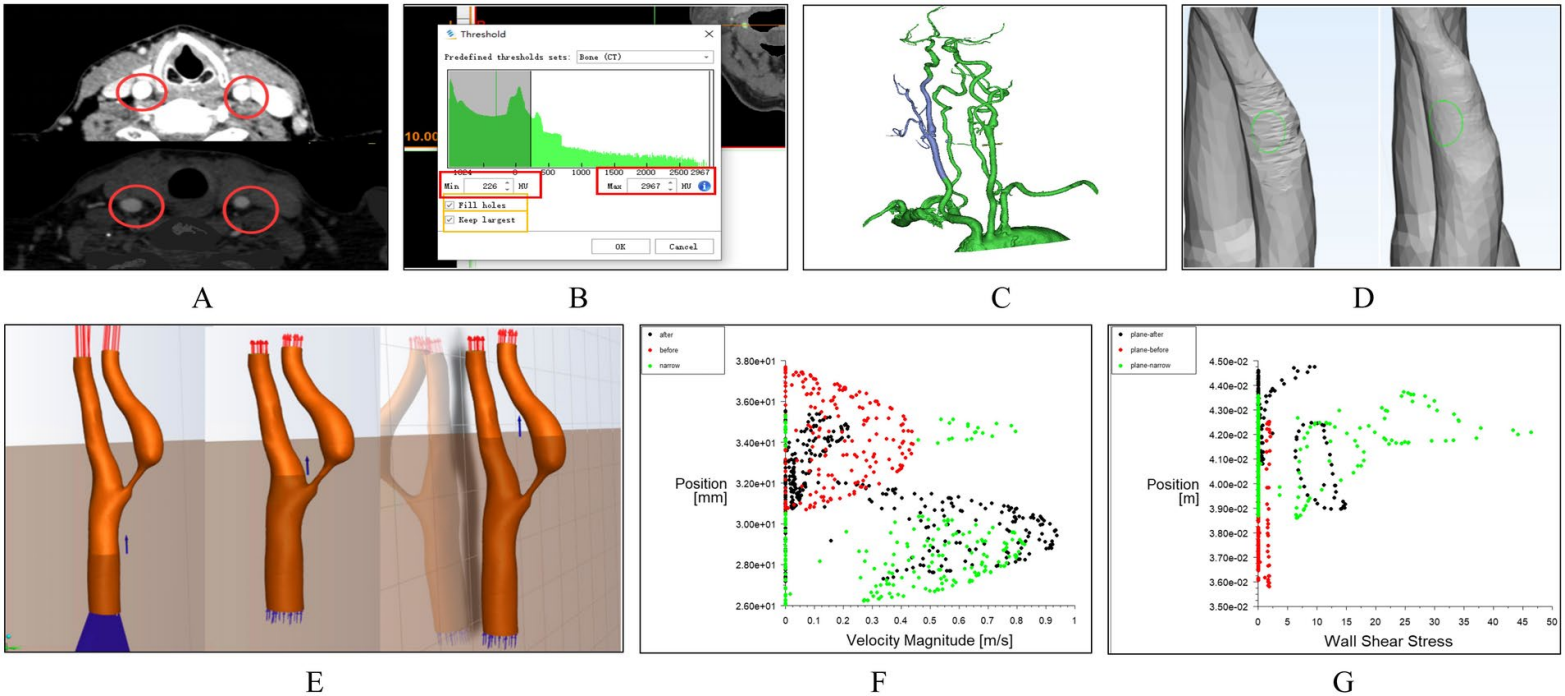
Workflow of carotid artery hemodynamics modeling. **(A)** Grayscale processing of medical images. **(B)** Threshold selection for image segmentation. **(C)** Construction of the 3D model of the carotid artery. **(D)** Removal of unrelated vascular structures and surface processing. **(E)** Simulation using the k-epsilon turbulence model. **(E)** Slicing and Labeling of Vascular Sections. **(F)** Scatter plots of velocity distribution. **(G)** Scatter plots of wall shear stress distribution.

Mimics Medical software (version 21.0, Materialise) was used to segment, reconstruct, and smooth the carotid CTA images, specifically focusing on the CCA, ICA, and ECA. Initially, medical images were gray-scaled (Figure 1A), and an appropriate threshold was chosen for image segmentation (Figure 1B), generating a 3D model (Figure 1C). Unrelated vascular structures were then removed from the 3D model, followed by overall smoothing (Figure 1D). To ensure the geometric fidelity of the arterial model, an isotropic voxel size was maintained during segmentation, allowing for high-resolution reconstruction of the vessel’s morphology. Subsequently, Materialise 3-matic software (version 13.0, X64) was used for further local smoothing of the reconstructed 3D model surface and for resection and labeling of vascular sections (Figure 1E). The vessel’s wall thickness was uniformly set based on the average thickness observed in the patient cohort, acknowledging that individual variations might exist but aiming for a standardized model. Error detection and repair were performed on the model, and the surface and volume mesh were exported for hemodynamic simulation using ANSYS-FLUENT (2022 R1).

During simulation, the k-epsilon turbulence model was utilized to replicate the turbulent behavior of the viscous fluid. This model is particularly suitable for boundary layer flows and general engineering flows, making it a fitting choice for simulating blood flow within vessels. The model assumes steady-state conditions for the blood flow, an approximation justified by the clinical observation of stable hemodynamics post-surgery. In the carotid artery model, the vessel walls were treated as rigid, no-slip boundaries. The blood was modeled as an incompressible Newtonian fluid with a density of 1,060 kg/m^3^ and a dynamic viscosity of 0.003 kg/(m·s). The input and output conditions were established based on CU parameters, ensuring that the boundary conditions reflect the actual physiological state of the patient. Specifically, velocity profiles were applied at the inlet, derived from the peak systolic and end diastolic velocities measured by ultrasound, while a constant pressure boundary condition was set at the outlet. Post-processing of the simulation results was conducted using CFD-Post (2022 R1) software. Cross-sections were added at different locations along the carotid artery, including pre-stenotic, post-stenotic, and stenotic regions (Figure 1E). From these sections, scatter plots of velocity distribution (Figure 1F) and wall shear stress (WSS) distribution (Figure 1G) were generated.

**Figure 2:**
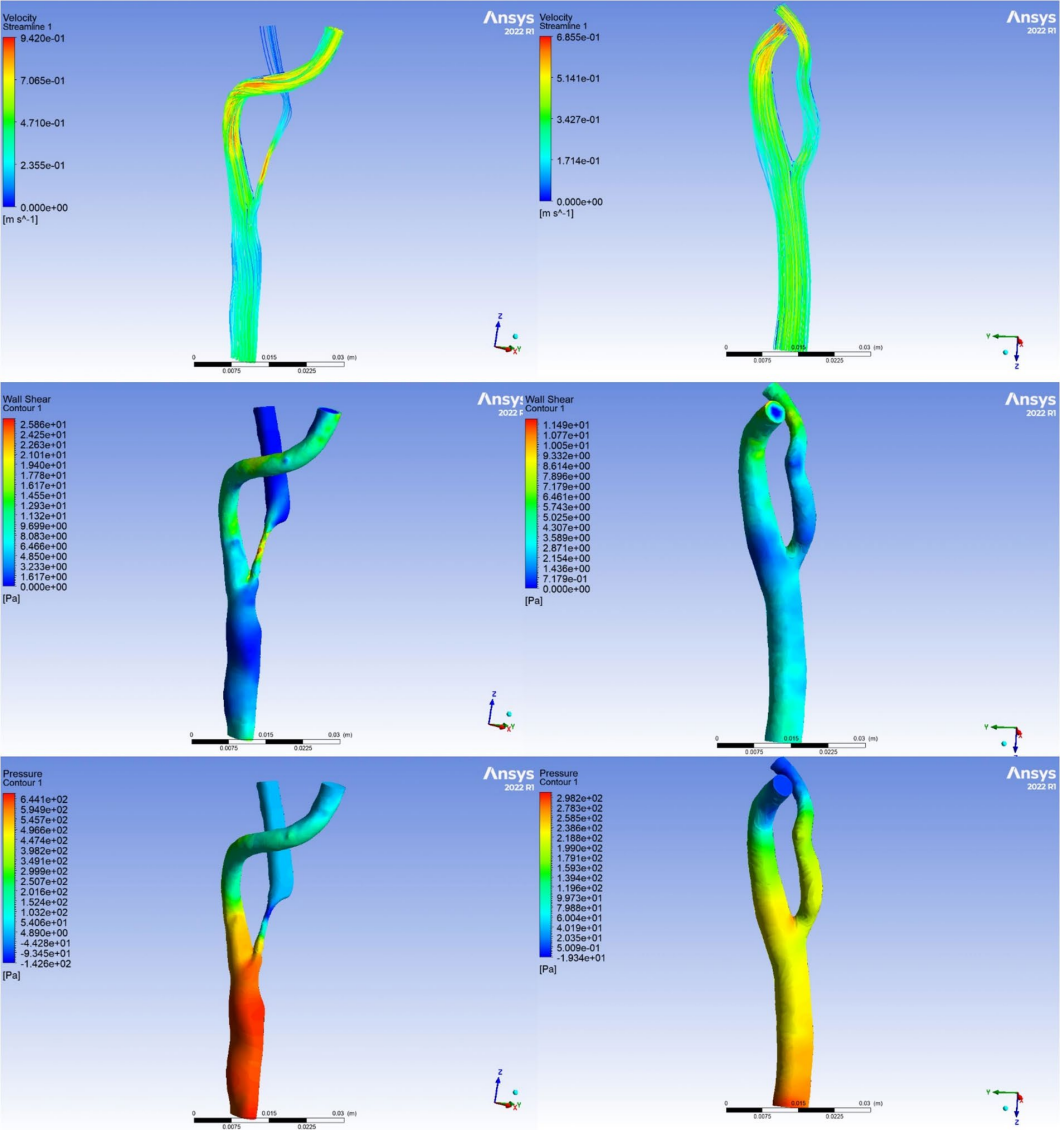
Visualization of hemodynamic parameters. The left panel illustrates preoperative hemodynamic parameters, representing baseline conditions. The right panel depicts postoperative hemodynamic parameters, demonstrating the alterations observed subsequent to the carotid endarterectomy procedure.

The parameters derived from the CFD model, including TP, WSS, PR, and WSSR, were utilized to quantify the relative variations in pressure and shear stress at each site of carotid stenosis. Figure 2 illustrates the comparison of hemodynamic parameters before and after CEA, with the preoperative images on the left and postoperative images on the right. The first row presented the velocity streamline distributions, depicting blood flow velocities. The second row showed the WSS distributions, while the third row displayed the total pressure distributions. TP denotes the overall pressure distribution along the x-axis of the ICA, while WSS represents the shear stress distribution along the y-axis. PR is defined as the ratio of pressure after stenosis to pressure before stenosis, and WSSR is calculated as the ratio of WSS at the stenosis site to WSS before stenosis. Changes in hemodynamic parameters pre- and post-surgery were calculated using the following formulas: ΔPR = (preoperative PR − postoperative PR) / preoperative PR × 100%, ΔTP = (preoperative TP − postoperative TP) / preoperative TP × 100%, ΔWSS = (preoperative WSS − postoperative WSS) / preoperative WSS × 100%, ΔWSSR = (preoperative WSSR − postoperative WSSR) / preoperative WSSR × 100%.

### Statistical analysis

Statistical analysis and visualization were conducted using SPSS 23.0 and R software (version 4.3.2). Continuous variables were summarized as mean ± standard deviation (SD) for normally distributed data and as median and interquartile range (IQR) for skewed data. Categorical variables were presented as frequencies and percentages. To compare hemodynamic parameters before and after CEA surgery, a paired *t*-test was used if variance homogeneity was satisfied; otherwise, the Wilcoxon signed-rank test was applied. Pearson correlation analysis identified factors linked to cerebral perfusion changes. To reduce the risk of multicollinearity and overfitting, variables with a *p* value under 0.01 in the univariate analysis were included in the multivariate logistic regression model as an initial step to identify key factors associated with CH (19). The predictive value of multimodal monitoring and single monitoring for cerebral perfusion was assessed using ROC curve analysis. Results were considered significant if *p* was less than 0.05.

## Results

### Baseline characteristics of CEA patients

This study included fifty-six patients who underwent CEA treatment. Baseline demographics, carotid lesion characteristics, and serum lipid levels are summarized in Table 1. The mean age of the patients was 69.21 years with a male predominance of 71%. Hypertension was present in 84% of patients, diabetes in 59%, hyperlipidemia in 63%, smoking in 75%, alcohol use in 38%, and coronary artery disease in 16%. The median levels of TG, TC, LDL-C, and HDL-C were 3.57 mmol/L, 1.85 mmol/L, 1.03 mmol/L, and 1.31 mmol/L, respectively. Left internal carotid artery (LICA) stenosis was observed in 45% of the patients, while 61% presented with symptoms. The median severity of stenosis was 76%. The mean length of hospital stay was 17.20 days, and the median hospitalization cost was 54,077 RMB (IQR: 52,154–60,968).

**Table 1:** Baseline clinical and demographic characteristics of patients.

Parameters	Multimodal group (*n* = 56)
Age, years	69.21 ± 8.19
Male, *n* (%)	40 (71%)
Disease history, *n* (%)	
Hypertension	47 (84%)
Diabetes mellitus	33 (59%)
Hyperlipidemia	35 (63%)
Smoking	42 (75%)
Alcohol	21 (38%)
Coronary heart disease	9 (16%)
Lipid levels, mmol/L	
TG	3.57 (3.05–4.19)
TC	1.85 (1.47–2.35)
LDL-C	1.03 (0.88–1.38)
HDL-C	1.31 (1.00–1.68)
Lesion	
LICA, *n* (%)	25 (45%)
RICA, *n* (%)	31 (55%)
Symptomatic ICA, *n* (%)	34 (61%)
Stenosis severity, %	76.00 (70.00–83.20)
Medical resource	
Length of hospital stay, day	17.20 ± 5.49
Hospitalization expenses, RMB	54,077 (52154–60,968)

Fisher’s exact test; HDL-C, high-density lipoprotein cholesterol; IQR, interquartile range; ICA, internal carotid artery; RICA, right internal carotid artery; LICA, left internal carotid artery; LDL-C, low-density lipoprotein cholesterol; SD, standard deviation; TC, total cholesterol; TG, triglycerides.

### Comparison of hemodynamic parameters pre- and post-CEA

Significant changes in hemodynamic parameters were observed in patients following CEA treatment (Table 2). Specifically, postoperative measurements of CU showed a significant decrease in MFV [1.05 cm/s (0.86–1.45) vs. 0.37 cm/s (0.27–0.44)], PSV [2.05 (1.67–2.92) cm/s vs. 0.66 (0.48–0.79) cm/s], EDV [0.52 cm/s (0.39–0.77) vs. 0.21 cm/s (0.17–0.25)], and RI [0.74 (0.67–0.77) vs. 0.66 (0.58–0.71)] (*p* < 0.001 for all). In contrast, ipsilateral CBF significantly increased postoperatively (38.49 ± 4.35 mL/100 g/min vs. 54.71 ± 9.70 mL/100 g/min, *p* < 0.001), accompanied by an increase in contralateral CBF (41.15 ± 4.00 mL/100 g/min vs. 43.25 ± 3.29 mL/100 g/min, *p* = 0.003). rCBF also increased significantly (0.95 ± 0.12 vs. 1.27 ± 0.26, *p* < 0.001). For rSO₂, ipsilateral rSO₂ increased significantly postoperatively (*p* = 0.013), while no significant change was observed in contralateral rSO₂ (*p* = 0.861). Furthermore, analysis of hemodynamic parameter change rates revealed substantial decreases in MFV, WSS, WSSR, and TP following CEA, with WSS showing the largest median reduction of 61.60% (IQR: 44.63–82.56%). Conversely, parameters such as CBF, PR, and rSO₂ showed increases, with PR demonstrating the largest median increase at 36.06% (IQR: 11.62–62.34%).

**Table 2:** Hemodynamic parameters of patients underwent CEA.

Hemodynamic parameters	Pre-CEA	Post-CEA	*p*-value
Carotid flow velocities			
PSV at ICA, cm/s	2.05 (1.67–2.92)	0.66 (0.48–0.79)	<0.001
EDV at ICA, cm/s	0.52 (0.39–0.77)	0.21 (0.17–0.25)	<0.001
MFV at ICA, cm/s	1.05 (0.86–1.45)	0.37 (0.27–0.44)	<0.001
RI (PSV-EDV/PSV)	0.74 (0.67–0.77)	0.66 (0.58–0.71)	<0.001
CT perfusion parameters, mL/100 g/min			
Ipsilateral CBF	38.49 ± 4.35	54.71 ± 9.70	<0.001
Contralateral CBF	41.15 ± 4.00	43.25 ± 3.29	0.003
rCBF	0.95 ± 0.12	1.27 ± 0.26	<0.001
Regional cerebral oxygen saturation, %			
Ipsilateral rSO_2_	69.21 ± 3.657	70.77 ± 4.892	0.060
Contralateral rSO_2_	70.82 ± 4.217	70.68 ± 4.361	0.861
Computational fluid-mechanical parameters			
TP	278 (141–601)	167 (98–278)	0.002
PR	0.63 (0.46–0.75)	0.79 (0.71–0.95)	<0.001
WSS	1.47 (0.57–1.84)	0.57 (0.36–1.29)	<0.001
WSSR	2.19 (1.26–3.57)	1.70 (1.17–3.12)	<0.001

Rates of hemodynamic parameters change	IQR
ΔCBF	35.50% (23.25–49.75%)
ΔMFV	41.56% (17.22–56.87%)
ΔrSO_2_	2.50% (1.00–5.00%)
ΔTP	26.38% (6.10–67.38%)
ΔPR	36.06% (11.62–62.34%)
ΔWSS	61.60% (44.63–82.56%)
ΔWSSR	39.31% (23.43–54.20%)

CBF, cerebral blood flow; CEA, carotid endarterectomy; EDV, end diastolic velocity; ICA, internal carotid artery; IQR, interquartile range; MFV, mean flow velocity; PR, translesional pressure ratio; PSV, peak systolic velocity; rCBF, relative cerebral blood flow; RI, resistance Index; rSO_2_, cerebral regional oxygen saturation; TP, total pressure; WSS, wall shear stress; WSSR, wall shear stress ratio; Δ, change rate.

### Correlation between changes in hemodynamic parameters and cerebral perfusion

**Figure 3:**
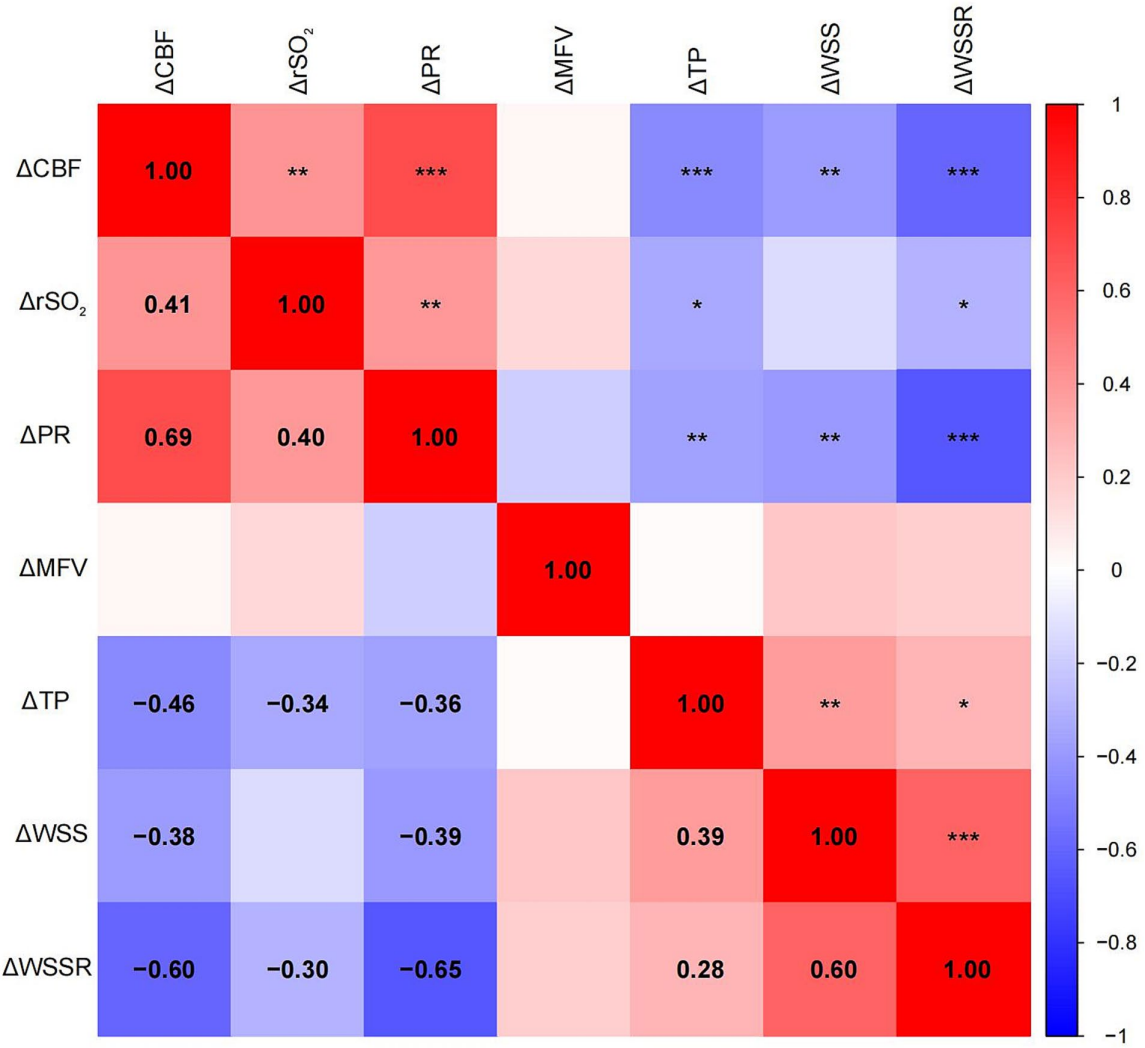
Heatmap of the Pearson correlation matrix between cerebral perfusion changes and mechanical parameter changes. The spectrum of colors denotes varying degrees of correlation, with blue representing negative correlation and red representing positive correlation. Significant correlations were indicated by statistical significance levels of * *p* < 0.05, ** *p* < 0.01, and *** *p* < 0.001.

Pearson correlation analysis revealed significant associations between ΔCBF and various hemodynamic parameters (Figure 3). ΔCBF was positively correlated with ΔPR (r = 0.69, *p* < 0.001) and ΔrSO₂ (r = 0.41, *p* < 0.01), and negatively correlated with ΔWSSR (r = −0.60, *p* < 0.001), ΔTP (r = −0.46, *p* < 0.001), and ΔWSS (r = −0.38, *p* < 0.01). No significant correlation was found between ΔCBF and ΔMFV (r = −0.07, *p* = 0.782). No significant correlation was found between ΔCBF and ΔMFV (r = −0.07, *p* = 0.782). These findings indicated that postoperative changes in cerebral perfusion were closely associated with variations in PR, rSO₂, TP, WSS, and WSSR. Careful regulation of these hemodynamic parameters is essential to minimize complications associated with excessive changes in cerebral perfusion.

### Factors associated with CH

Among the cohort of CEA patients, nine patients developed CH in the postoperative period. One patient suffered seizures within 24 h post-surgery, two patients reported significant headaches within 3 days, and the remaining six were asymptomatic. Variables with a *p*-value less than 0.01 in the univariate analysis, including pre-PR, pre-WSSR, pre-WSS, ΔPR, and ΔWSSR, were incorporated into the multivariate logistic regression model to identify independent predictors of CH. Although pre-WSS and ΔWSSR were significant in the univariate analysis, they did not maintain statistical significance after adjustment, indicating that their predictive effects may be confounded by or secondary to other factors. In contrast, pre-WSSR (OR: 2.353, 95% CI: 1.821–6.747, *p* = 0.011) and ΔPR (OR: 1.009, 95% CI: 1.002–1.025, *p* = 0.015) were identified as independent predictors of CH (Table 3).

**Table 3:** Univariate and multivariate logistic regression analyses of fluid-mechanical parameters associated with CH.

Factors	Univariable		Factors	Multivariable	
	OR (95% CI)	*p*-value		OR (95% CI)	*p*-value
Pre-TP	1.001 (1.000–1.002)	0.029*	Pre-PR	0.841 (0.347–1.841)	0.974
Pre-PR	0.005 (0.001–0.046)	0.003**	Pre-WSS	0.988 (0.500–1.952)	0.943
Pre-WSS	0.936 (0.898–0.975)	0.001**	Pre-WSSR	2.353 (1.821–6.747)	0.011*
Pre-WSSR	3.828 (1.639–8.940)	0.002**	ΔPR	1.009 (1.002–1.025)	0.015*
ΔTP	0.966 (0.939–0.993)	0.014*	ΔWSSR	0.936 (0.796–2.067)	0.163
ΔPR	1.017 (1.006–1.028)	0.002**			
ΔWSS	0.945 (0.900–0.991)	0.020*			
ΔWSSR	1.068 (1.026–1.114)	0.001**			

CH, cerebral hyperperfusion; CI, confidence interval; OR, odds ratio. * *p* < 0.05, ** *p* < 0.01.

### Predictive value of multimodal and single-modality monitoring for CH

**Figure 4:**
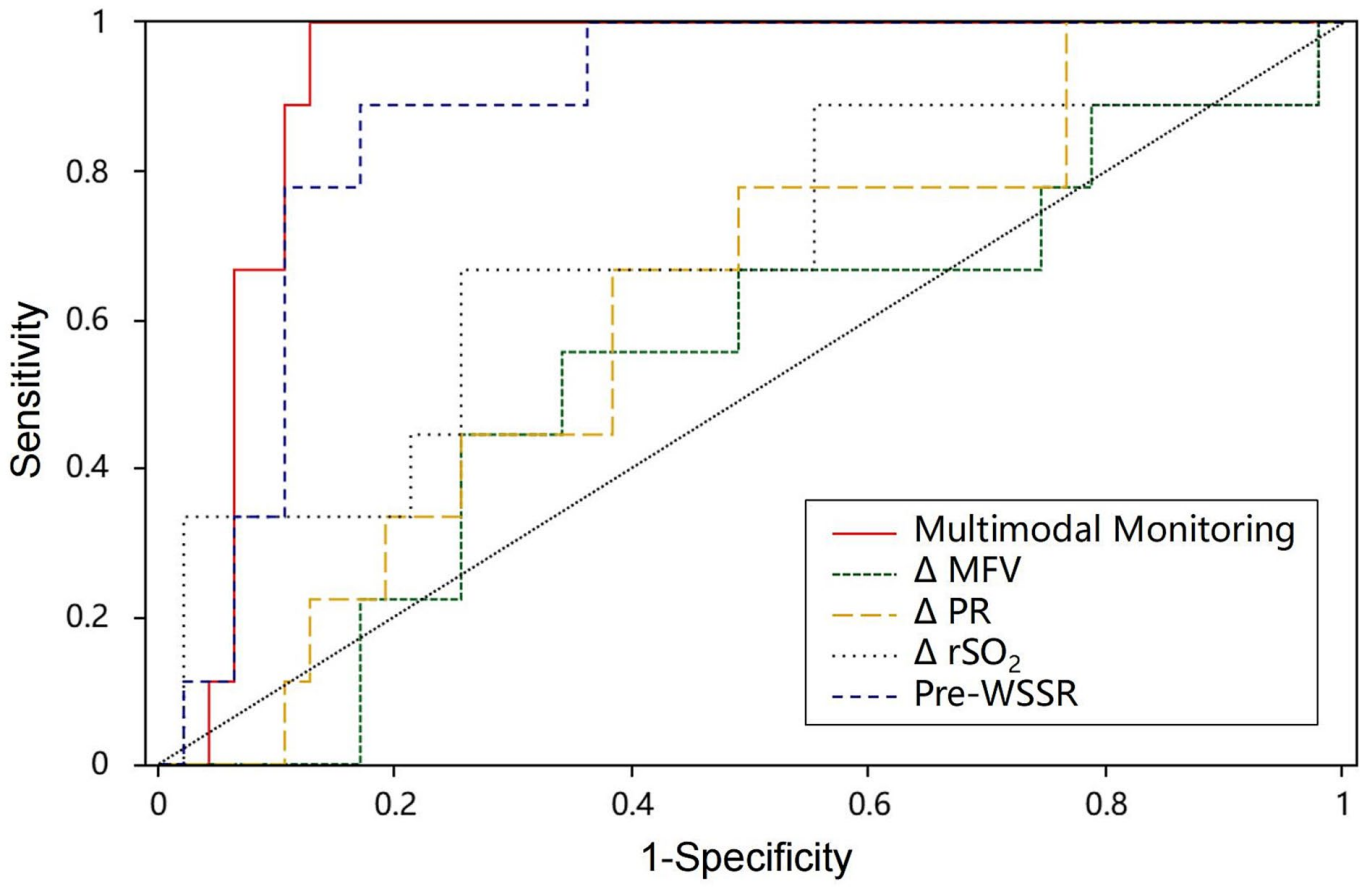
The ROC curve of post-CEA CH. AUC, area under the curve; Multimodal monitoring, a combination of Pre-WSSR, *Δ*PR, ΔMFV, and ΔrSO₂; ROC curve, receiver operating characteristic curve.

The predictive performance of multimodal and single-modality monitoring for post-CEA CH is summarized in Table 4. Among single-modality metrics, Pre-WSSR demonstrated the highest predictive value, with an AUC of 0.850 (95% CI: 76.55–95.53%), sensitivity of 0.889, and specificity of 0.773 (*p* < 0.01), establishing it as the most reliable single predictor. ΔPR showed moderate predictive power, with an AUC of 0.664 (95% CI: 58.72–74.88%), sensitivity of 0.667, and specificity of 0.727 (*p* < 0.01). In contrast, ΔMFV and ΔrSO₂ exhibited lower predictive accuracy, with AUCs of 0.573 and 0.681, respectively, and neither achieving statistical significance (*p* = 0.489 and *p* = 0.088, respectively). Notably, multimodal monitoring, combining Pre-WSSR, ΔPR, ΔMFV, and ΔrSO₂, demonstrated the highest predictive performance, with an AUC of 0.922 (95% CI: 85.00–99.40%), sensitivity of 0.995, and specificity of 0.872 (*p* < 0.01). The ROC curves in Figure 4 further illustrate the superior diagnostic performance of multimodal monitoring compared to single-modality approaches. The integration of multiple hemodynamic and oxygenation parameters provides a more robust and reliable strategy for predicting post-CEA CH, underscoring its potential clinical utility for early detection and risk stratification.

**Table 4:** Predictive value of monitoring methods for post-CEA CH.

Variable	AUC	Cut-off	95%CI	Sensitivity	Specificity	*p*-value
Combined	0.922	0.957	85.00–99.40%	0.995	0.872	<0.01
Pre-WSSR	0.850	2.800	76.55–95.53%	0.889	0.773	<0.01
ΔPR	0.664	0.825	58.72–74.88%	0.667	0.727	<0.01
ΔMFV	0.573	0.012	36.60–78.00%	0.556	0.660	0.489
ΔrSO_2_	0.681	0.719	47.00–89.20%	0.667	0.745	0.088

AUC, area under curve; CH, cerebral hyperperfusion; ROC curve, receiver operating characteristic curve.

## Discussion

CEA remains a cornerstone intervention for preventing stroke in patients with carotid artery stenosis. However, the procedure carries the risk of perioperative complications due to hemodynamic changes, which can lead to severe neurological outcomes (2, 20). This study first integrated multimodal monitoring techniques with CFD analysis to comprehensively assess hemodynamic changes during the perioperative period in CEA patients. Factors contributing to changes in cerebral perfusion were explored, and further investigation identified risk factors for post-CEA CH and evaluated their predictive value.

### Hemodynamic changes post-CEA

Our findings demonstrated significant alterations in hemodynamic parameters post-CEA. CU measurements showed a substantial reduction in MFV, PSV, EDV, and RI in the ipsilateral ICA, indicative of improved hemodynamic flow and reduced stenosis severity. These changes were complemented by increases in ipsilateral CBF and rCBF, reflecting enhanced perfusion in the middle cerebral artery territory. The observed increase in rSO2 on the operated side post-CEA may indicate effective restoration of cerebral perfusion and oxygenation. Previous studies (21–23) have corroborated these findings, validating the enduring relevance of these observations in current clinical practice.

Notably, the findings from the CFD analysis revealed that postoperative reductions in TP, WSS, and WSSR, along with an increase in translesional PR, reflected the biomechanical mechanisms underlying hemodynamic alterations after CEA. Specifically, the removal of plaque during CEA lowered blood flow resistance and TP, reducing the force on vessel walls and indicating less stenosis and cardiac workload. In patients with arterial stenosis, high preoperative translesional PR usually signals a severe pressure gradient and restricted blood flow due to significant carotid artery stenosis (24, 25). An increase in PR after CEA indicates improved pressure gradient across the previously narrowed segment, signaling successful revascularization. Monitoring TP evaluates CEA’s effectiveness in reducing carotid artery hemodynamic stress, while PR assesses restored cerebral blood flow, helping prevent complications like cerebral hyperperfusion syndrome.

WSS is the force exerted tangentially by blood flow on the arterial wall, influenced by blood viscosity and shear rate. The pulsatile shear forces generated by each heartbeat directly affect endothelial function (26). Following CEA, reduced WSS indicates less mechanical stress on the vessel wall, while decreased WSSR suggests a more uniform ICA geometry and shear stress distribution (27). This even distribution of shear stress can lower turbulent forces, potentially reducing the risk of endothelial damage and atherosclerotic plaque formation (28). Our findings demonstrated the significant impact of CEA on improving hemodynamic parameters, consequently mitigating vascular stress and potential cardiovascular hazards. These insights underscored the importance of monitoring TP, WSS, WSSR, and PR in assessing the efficacy of CEA and guiding postoperative management strategies to optimize patient outcomes and prevent complications.

### Correlation of hemodynamic changes with cerebral perfusion changes

Pearson correlation analysis preliminary revealed significant associations between ΔCBF and multiple factors (ΔPR, ΔrSO2, ΔTP, ΔWSS, and ΔWSSR). Positive correlations were observed between ΔCBF and ΔPR as well as ΔrSO2, suggesting a potential link between increased postoperative hyperperfusion and these parameters. Conversely, negative correlations were found between ΔCBF and ΔTP, ΔWSS, and ΔWSSR, indicating that increases in these parameters following CEA may be beneficial in preventing hyperperfusion events. These correlations highlight the intricate relationship between hemodynamic changes post-surgery and cerebral perfusion.

In a multivariable logistic regression analysis, both preoperative WSSR and ΔPR were identified as independent predictors of post-CEA CH. The interpretation of these factors as independent risk factors for hyperperfusion may be linked to their respective physiological mechanisms. Firstly, elevated preoperative WSSR may indicate abnormal hemodynamic distribution in stenotic areas, potentially leading to uneven shear forces on the local vascular endothelium. High WSSR has the potential to activate thrombosis pathways and boost matrix metalloproteinase expression. Post-vascular reconstruction, altered blood flow, especially after stenosis removal, can trigger endothelial cells to release cytokines, increasing permeability and changing local hemodynamics, which promotes hyperperfusion (29). Additionally, ΔPR reflects changes in hemodynamics in the stenotic region pre- and post-operatively (20). Elevated preoperative PR suggested significant pressure gradients within the stenotic area, indicating hemodynamic imbalance before surgery. Successful CEA improved hemodynamics in the stenotic region, leading to a significant reduction in PR, reflecting redistributed and restored blood flow, thereby ensuring adequate cerebral perfusion. This highlights the importance of cerebrovascular autoregulation in modulating cerebral blood flow postoperatively.

### Clinical value of multimodal monitoring based on CFD

In recent years, there has been a growing interest in the influence of biomechanical factors on hemodynamics. Researchers have investigated various aspects, including WSS, intramural stress, and particulate transport, resulting in the development of multiple WSS-related biomechanical indicators (30). Observing the evolution of these parameters revealed that current biomechanical metrics are predominantly focused on characterizing the shear stress encountered by endothelial cells and assessing the initial stages of atherosclerosis (31). Nevertheless, there is limited research on the long-term effects of therapeutic interventions. Given that, our study provides a preliminary exploration of the application value of CFD-based multimodal monitoring in the perioperative management of patients undergoing CEA.

Our study highlights the significant predictive value of multimodal monitoring in identifying the risk of post-CEA CH, offering a substantial advantage over single-modality approaches. Multimodal monitoring, which integrates Pre-WSSR, ΔPR, ΔMFV, and ΔrSO₂, achieved the highest predictive accuracy with an AUC of 0.922, sensitivity of 0.995, and specificity of 0.872. This demonstrates that the combination of CU, CDF, and oxygenation parameters provides a comprehensive assessment of cerebral perfusion dynamics, enabling early and reliable detection of CH. Among the single-modality metrics, pre-WSSR stood out with the highest predictive performance (AUC = 0.850). However, other single-modality metrics, such as ΔMFV and ΔrSO₂, showed relatively lower predictive power, emphasizing the limitations of relying on individual parameters to assess the multifactorial nature of CH. The integration of multiple metrics in multimodal monitoring compensates for these limitations, as it accounts for the complex interactions among cerebral blood flow, vascular resistance, and oxygenation.

Nevertheless, it is crucial to consider its impact on hospital length of stay and healthcare costs. In this study, the average hospital stay for patients was 17.20 days, with a median hospitalization cost of 54,077 RMB. According to the 2023 report by the Organisation for Economic Co-operation and Development, the average length of hospital stay among member countries is approximately 7.5 days, with relatively lower healthcare expenses (32). These findings indicate that the hospital stay and costs observed in this study are significantly higher than global averages. The prolonged hospital stay and increased costs associated with multimodal monitoring may primarily result from the requirement for advanced equipment and technology, which raises healthcare expenses, as well as the need for extended monitoring durations to collect sufficient data, leading to longer hospitalizations. Addressing the growing burden of healthcare costs is a shared responsibility. In the current landscape of accountable care, only treatments that are both highly effective and cost-efficient will gain acceptance in the healthcare system. Future studies should focus on evaluating the cost-effectiveness of multimodal monitoring to determine its applicability across various healthcare settings. Additionally, efforts should be made to optimize monitoring protocols to reduce unnecessary hospital stays and expenses, ultimately improving both the overall patient experience and resource utilization.

### Limitations

Several limitations should be acknowledged in interpreting our findings. Firstly, the study’s single-center design and relatively small sample size may constrain the external validity of our findings. A larger, multi-center study would provide more robust data and improve the external validity of our conclusions. Firstly, the study’s single-center design and small sample size, though comparable to similar CFD studies, may limit the generalizability of our findings. Increasing sample sizes is often constrained by the complexity and cost of high-fidelity imaging and detailed simulations. Consequently, the small sample size might restrict the detection of subtle variations across different patient populations. A larger, multi-center study would provide more robust data and improve the external validity of our conclusions. Additionally, the assumptions made in our CFD models, such as considering blood as a Newtonian fluid and treating vessel walls as rigid, may impact the accuracy of our simulations. Blood exhibits non-Newtonian behavior, and vessel walls are compliant; thus, idealizing these properties could lead to discrepancies between our model predictions and actual physiological conditions. Future studies should aim to incorporate more realistic assumptions to improve model fidelity. Lastly, our analysis focused primarily on the biomechanical aspects of hemodynamics, potentially overlooking other factors such as biochemical interactions and cellular responses. A more comprehensive approach that includes these factors would provide a more holistic understanding of the pathophysiological processes involved.

## Conclusion

CFD-based multimodal monitoring effectively identified cerebral perfusion changes and risk factors for CH in CEA patients, with superior predictive accuracy compared to single-modality methods. This approach offers valuable insights for risk stratification and personalized perioperative management. Nevertheless, further validation through large-scale, multicenter studies is required to establish its clinical utility, particularly in evaluating its cost-effectiveness and applicability across diverse healthcare settings.

## Data Availability

The raw data supporting the conclusions of this article will be made available by the authors, without undue reservation.
